# Effect of sterilization on 3-point dynamic response to in vitro bending of an Mg implant

**DOI:** 10.1186/s40824-021-00207-9

**Published:** 2021-04-06

**Authors:** Luis Humberto Campos Becerra, Marco Antonio Loudovic Hernández Rodríguez, Raúl Lesso Arroyo, Hugo Esquivel Solís, Alejandro Torres Castro

**Affiliations:** 1 Facultad de Ingeniería Mecánica y Eléctrica, Universidad Autónoma de Nuevo León (UANL), Pedro de Alba S/N, ciudad universitaria, San Nicolás de los Garza, NL Mexico; 2 Departamento de Ingeniería Mecánica., Biomecánica, Instituto Tecnológico de Celaya (ITC), Av. Tecnológico Esq. A. García Cubas S/N Col. Bonfil, Celaya, 38010 Guanajuato, CP Mexico; 3 Centro de Investigación y Asistencia en Tecnología y Diseño del Estado de Jalisco (CIATEJ), Av. Normalistas No.800, Colinas de la normal C.P, 44270 Guadalajara, Jalisco Mexico

**Keywords:** Intramedullary implant, Fatigue resistance, Morphological characterization, Biological evaluation, Toxicity

## Abstract

**Background:**

The aim of the study is to characterize a biomedical magnesium alloy and highlighting the loss of mechanical integrity due to the sterilization method. Ideally, when using these alloys is to delay the onset of degradation so that the implant can support body loads and avoid toxicological effects due to the release of metal ions into the body.

**Methods:**

Standardized procedures according to ASTM F-1264 and ISO-10993-5 were used, respecting detailed methodological controls to ensure accuracy and reproducibility of the results, this testing methodology is carried out in accordance with the monographs of the Pharmacopoeia for the approval of medical devices and obtaining a health registration. An intramedullary implant (IIM) manufactured in magnesium (Mg) WE43 can support loads of the body in the initial period of bone consolidation without compromising the integrity of the fractured area. A system with these characteristics would improve morbidity and health costs by avoiding secondary surgical interventions.

**Results:**

As a property, the fatigue resistance of Mg in aggressive environments such as the body environment undergoes progressive degradation, however, the autoclave sterilization method drastically affects fatigue resistance, as demonstrated in tests carried out under in vitro conditions. Coupled with this phenomenon, the relatively poor biocompatibility of Mg WE43 alloys has limited applications where they can be used due to low acceptance rates from agencies such as the FDA. However, Mg alloy with elements such as yttrium and rare earth elements (REEs) have been shown to delay biodegradation depending on the method of sterilization and the physiological solution used. With different sterilization techniques, it may be possible to keep toxicological effects to a minimum while still ensuring a balance between the integrity of fractured bone and implant degradation time. Therefore, the evaluation of fatigue resistance of WE43 specimens sterilized and tested in immersion conditions (enriched Hank’s solution) and according to ASTM F-1264, along with the morphological, crystallinity, and biocompatibility characterization of the WE43 alloy allows for a comprehensive evaluation of the mechanical and biological properties of WE43.

**Conclusions:**

These results will support decision-making to generate a change in the current perspective of biomaterials utilized in medical devices (MDs), to be considered by manufacturers and health regulatory agencies. An implant manufactured in WE43 alloy can be used as an intramedullary implant, considering keeping elements such as yttrium-REEs below as specified in its designation and with the help of a coating that allows increasing the life of the implant in vivo.

## Introduction

Today, the use of new biomaterials in the manufacturing of medical devices (MDs) in the world is of great relevance, as reducing the environmental impact of extracting and refining currently popular elements such as titanium (Ti) is an important goal [1]. Currently, the potential of magnesium (Mg) as a base material for alloying with essential and nutritional elements such as Ca, Zn, and REEs is being widely investigated [2–6]. The advantages and effects of its use have already been evaluated in vivo by different programs and organizations throughout the world [7–11]. Several studies have proposed using elements that provide mechanical resistance and improve properties such as the degradation and biocompatibility of Mg. As a result, modifications have been proposed to the committee of “biological and clinical evaluation of medical devices ISO/TC 194/WG5” concerning the Mg processed from smelting, suggesting increasing the extraction medium mentioned in ISO 10993-5: 2009 by 10 times, to allow the release of Mg ions in vitro to be maintained at acceptable levels in the rankings cytotoxicity. This modification would be part of an eventual change in ISO 10993-15 which would favor the use of Mg in MDs, particularly in the context of materials designed to degrade in the body [10, 12–16]. Additionally, there are techno-surveillance programs and successful clinical cases where Mg has already been used as the base material for MDs [11, 17–21].

There are some medical devices manufactured based on Mg in the European market which have authorization for use in clinical trials issued by the Korean Food and Drug Administration (KFDA) [12, 19, 22, 23]. Notably, H. Sun et al. have considered Mg as part of the new generation of biomaterials for medical applications [10, 24–26]. Despite the excellent alternatives proposed for alloying elements that can be combined with Mg, elements such as Zn and REEs should be dosed [12, 20, 27], to avoid toxicological and pathophysiological problems [19]. In order to obtain an optimum point balancing the time of recovery for a fracture and the degradation of an implant, different dosages of elements and the refinement of grains during smelting must be tested and analyzed [27–29].

Despite all the work done in the field of mechanical and biological characterization of Mg alloys, an experimental evaluation of how an Mg alloy’s crystalline structure is related to its biocompatibility is still necessary. This work shows the mechanical characterization through mechanical tests established by American Society of Testing Materials (ASTM) for intramedullary nails, testing and measuring typical stress-strain curves at simple tension, simple flexion, and fatigue flexion. Furthermore, the morphological-crystalline characterization is shown through an evaluation by TEM, SEM-EDS, and XRD. Along with a simulation utilizing finite element method (FEM) to visualize the behavior of grain boundaries, this combinatorial approach presents mechanical tests, crystalline structure, and mechanical behavior of Mg alloys. Additionally, cellular interactions on the surface of the WE43 alloy are investigated for biocompatibility and a probable model for in vitro absorption is established. By furthering the understanding of the biological response of Mg, the biological value of Mg and better predict outcomes in clinical practice utilizing Mg-based alloys, proprioception about the use of degradable metallic materials is generated [10, 25, 30–32].

## Methods

### Materials

Mg WE43 base alloy (Yttrium: 3.1–3.7% wt; Lanthanum: 0.07–0.33% wt; Gadolinium: 1.7–1.8% wt; Neodymium: 2.2–2.5% wt; Copper: ≤ 0.16% wt; Nickel: ≤ 0.11% wt; Iron: ≤ 0.017% wt; Oxygen: ≤ 4.62% wt) foundry from the Pidgeon process in the condition of casting bars, is proposed for the manufacture of an intramedullary implant (IIM). The IIM described here has been tested following an experimental methodology according to international standards ASTM-F-1264, ISO10993, and additional morphological characterization.

### Preparation of Mg implants

Three specimens were manufactured according to ASTM B 557, of which the stress-strain curve was obtained following ASTM E8 to characterize the behavior of the alloy in simple tension to 0.2% deformation. The behavior of the alloy is described by the isotropic multi-linear curve showing homogeneity. Additionally, three specimens were prepared to perform a simple bending test according to section A1.4 of ASTM F-1264 with a diameter approximately equal to that proposed for the IIM to know the resistance to bending with a force-displacement curve.

### Sterilization

Two other specimens were machined to perform a flexural test under dynamic conditions to measure the loss of mechanical integrity of the material due to the degradation that originates on the surface of the material by sterilization and the enriched Hank’s solution. The Hank’s solution was obtained from Sigma Aldrich and was used according to ISO 10993-12. One of the specimens was sterilized using autoclave the method that most affects the surface of the Mg alloys [33]. All procedures were carried out under aseptic conditions and in a sterile environment, ensuring cell recovery, adherence, and progression towards the exponential growth phase [30]. Three WE43 alloy specimens were made and sterilized utilizing an autoclave followed by a 3-point simple bending test based on ASTM F 1264 to evaluate the flexural strength and to obtain the force-displacement curve of each specimen. A test was carried out in dynamic conditions to measure strength parameters vs. number of cycles of the WE43 alloy immersed in Hank’s balanced salts. The alloy was evaluated under fatigue conditions for two specimens according to ASTM F 1264. In preparation, these manufactured specimens were simply polished with 800 grit sandpaper and were not attacked chemically. Only one specimen was subjected to sterilization. Immersion tests were performed following ASTM-G31–72 [34, 35].

The technical requirements for the approval of MDs include the review of good manufacturing practices by a regulated body such as the FDA, in addition to this, scientific and technical information must be provided, in addition to a detailed label that implies the surgical technique and the description of functional components, parts, and structure. The list of the elements used in the alloy and their dispersion on the implant surface must be presented, the qualitative-quantitative formula must be declared per unit of measure and percentage dose, in addition to the raw materials and a study where the toxicity, safety, and efficacy characteristics of MDs are supported. Information should be provided on the sterilization process, control of the finished product and analytical methods, stability study, and techno vigilance. This work presents the experimental morphological evaluation applicable to implantable products, in addition to the preclinical studies of biocompatibility; the severity of autoclave sterilization is reported due to fatigue resistance and biological reactivity for class III products.

### Mechanical test

**Fig. 1:**
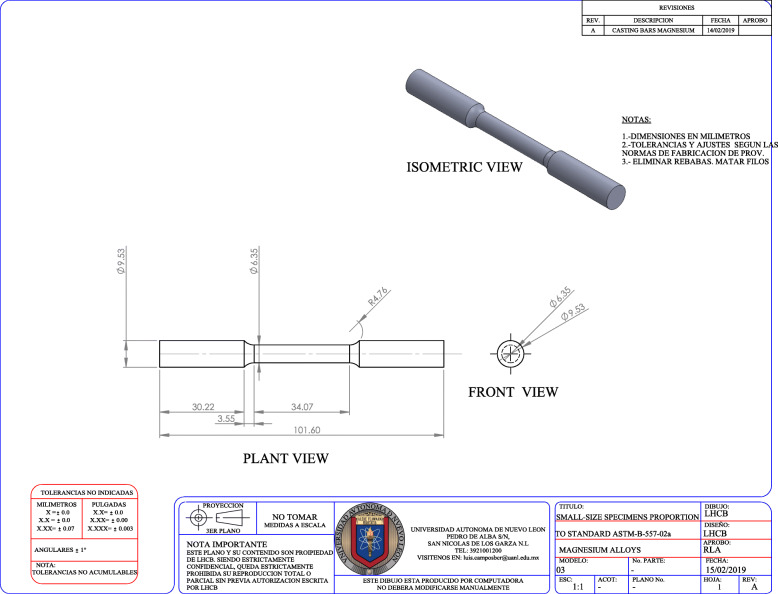
General dimensions of tension specimen.(mm)

The setup for stress tests was based on ASTM E8–04 (standard test methods for tension testing of metallic materials). Realized in three specimens with the configuration and dimensions established by ASTM B 557–02 (standard test methods of tension testing wrought and cast aluminum and magnesium - alloy products). These specimens were manufactured according to Fig. 1, where the geometry of the specimen to be tested is shown.

Table 1 shows the characteristics of the simple stress test applied to the three test specimens, where its stress-strain curve was obtained. The data acquisition software testXpert2 was used to acquire the maximum displacement of each of the tests and to record and plot the stress vs. strain curve.

**Table 1:** Simple stress test characteristics

Test feature or detail	Technical data.	Observations.
Test speed (velocity)	10 mm/min	-
Used machine.	Zwick/Roel z050, capacidad 50 kN	25 enero 2019 calibración
Cross section of the specimen.	31.66 mm ^2^	-
Specimen length.	101.60 mm	-
Specimen material.	WE43 Casting bars	Y 3.7-4.3, Zr 0.4-1.0, Gd 0-1.9, Nd 2.0-2.5, Cu ≤ 0.02, Ni ≤ 0.005, Fe ≤0.01, Mg balance
Average weight specimen.	0.01058 kg	-
Test temperature.	22 °C	-

**Fig. 2:**
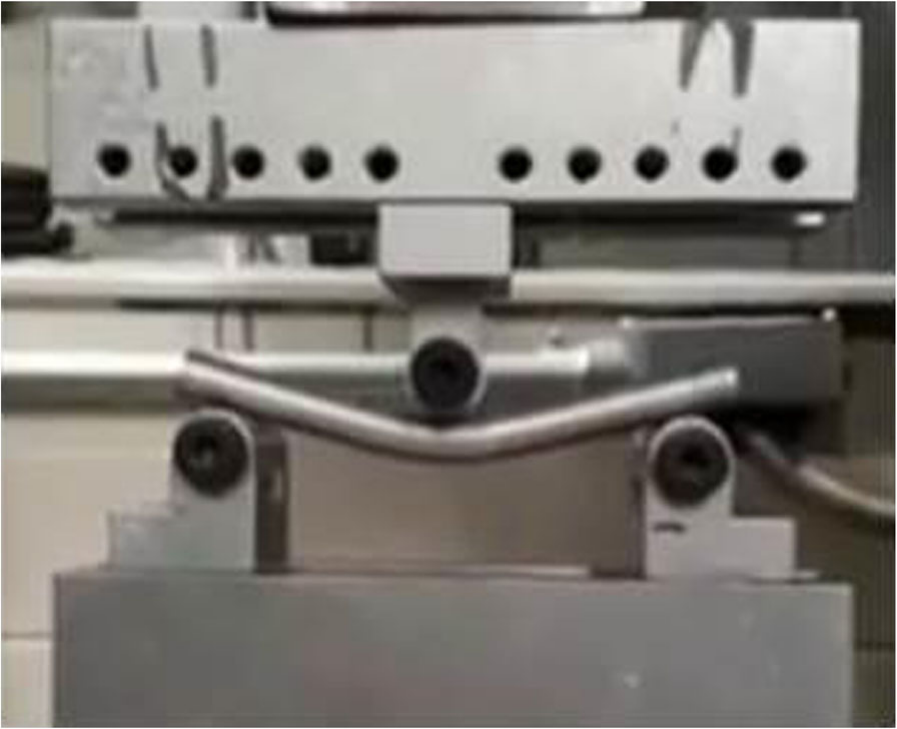
Test specimens (ASTM F1264–03) 3 pointing bending test scheme (A1.4, A4.2)

The setup for the 3-point simple bending tests was based on ASTM F1264–03 (standard specification and test methods for intramedullary fixation devices) Realized in three specimens with the configuration proposed in the procedure of section A1.4. These specimens were manufactured according to ASTM B 557–02. Figure 2 shows a specimen during the 3-point test. This applies to scenarios where it is necessary to know the resistance to bending when a nail is placed on two points and a load is applied at one point between them.

Three tests were performed where the force and displacement were obtained starting at a 0 N load and ending at the maximum deflection load for each specimen. The details of the test and materials are shown in Table 2. A VI data acquisition system with a LabVIEW R2017 interface was used to acquire the maximum displacement of each for the tests and to plot the force vs. displacement curve.

**Table 2:** Characteristics of 3-point bending test

Test feature or detail	Technical data.	Observations.
Distance between centers L.	90 mm	-
Test speed (velocity)	5 min/min	F-1264-03
Used machine	Instron 1011, capacity 5 kN	Jan 25, 2019 Mess.Serv.Metrological
Specimen cross section	71.48 mm ^2^	-
Specimen length.	114 mm	-
Specimen material.	WE43 Casting bars	Y 3.7-4.3, Zr 0.4-1.0, Gd 0-1.9, Nd 2.0-2.5, Cu ≤ 0.02, Ni ≤ 0.005, Fe ≤0.01, Mg balance
Average weight specimen	0.01511 kg	-
Test temperature.	27 °C	-

The system shown in Fig. 2 was used to perform the 3-point simple bending tests on specimens with a diameter of 9.54 mm and length of 114 mm. This test consists of two supports and a lower base to place the specimen, as well as an upper support mounted on the load cell of the Instron 1011 machine which applies the load during the test to the center of the specimen.

**Fig. 3:**
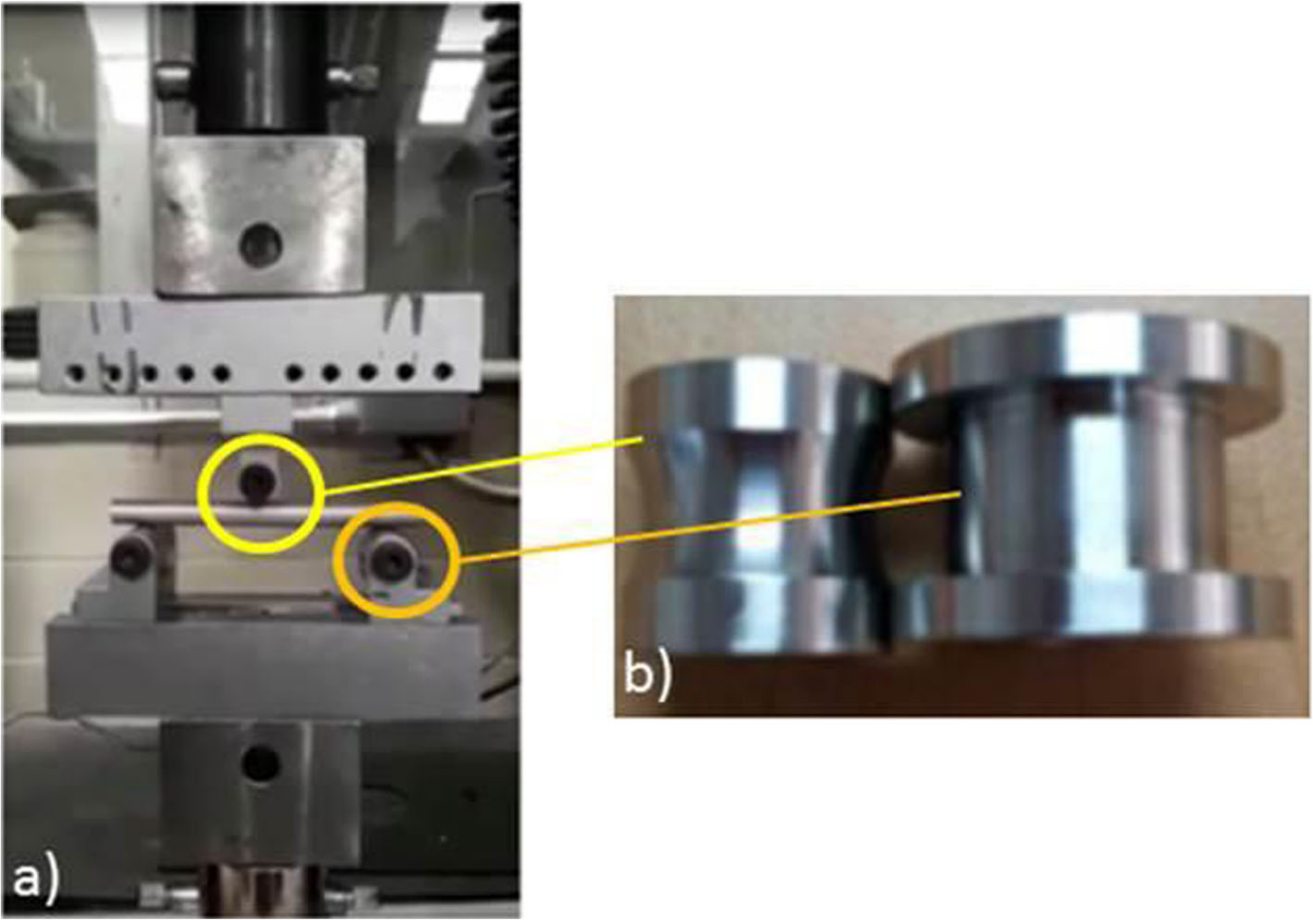
**a** System used in simple bending tests for the WE43 9.54ø × 114 mm specimen. **b** Load applicator rollers

A DAQ SB10 and a VI program were used to record force reached and maximum displacement of each of the tests, and then to record and plot the force vs. displacement curve. Figure 3 displays this system.

The results setup for the 3-point dynamic flexion tests was based on ASTM F1264–03 (standard specification and test methods for intramedullary fixation devices). This test was realized on two specimens with the configuration proposed in the procedure of section A1.4.

**Fig. 4:**
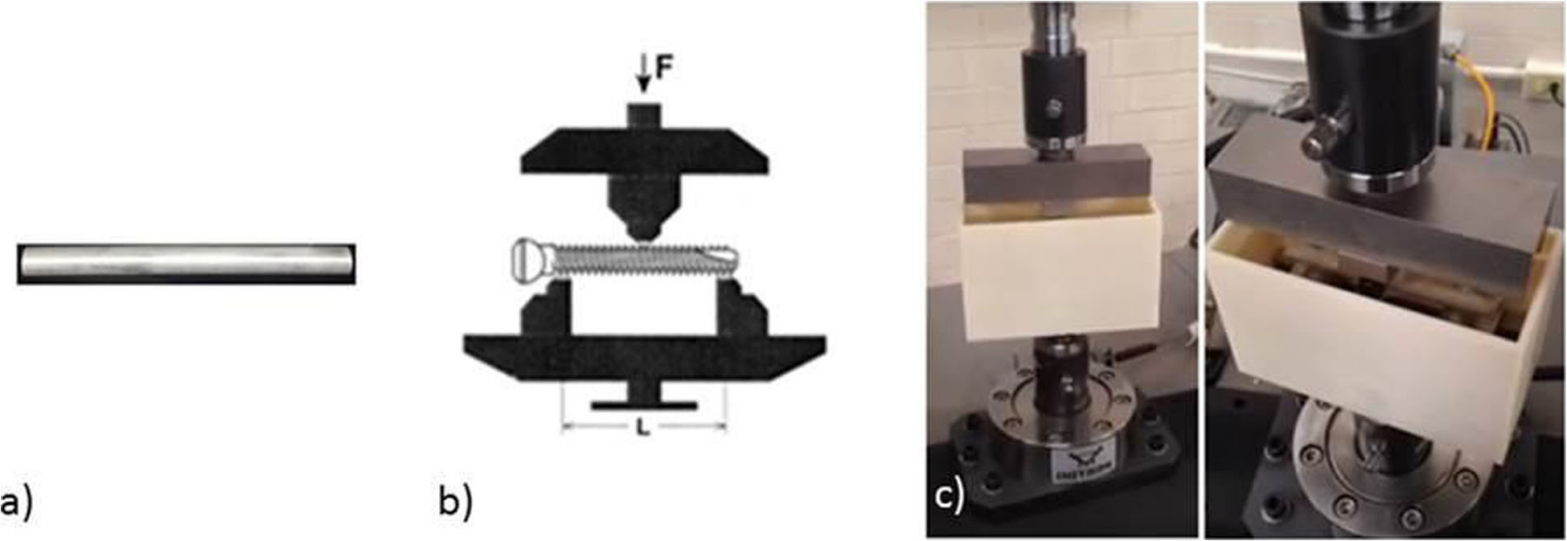
**a** Test specimen for fatigue flexion tests at 3 points. **b** System used to perform a fatigue flexion test. **c** Instron 1011 machine configuration with polypropylene chamber assembly for specimen immersion

These specimens were manufactured according to ASTM B 557–02 and are displayed in Fig. 4a. This test determines the resistance to flexion by fatigue when placing a nail on two points and applying a dynamic load at 1 point. The full setup is described in Fig. 4.

Two tests were performed where the force vs. number cycles were obtained from a 25% load amplitude (555 N). Data obtained from the simple flex test to stabilize the system, the details of the test and materials are shown in Table 3. Also, a VI data acquisition system with a LabVIEW R2017 interface was used to acquire the maximum force of each of the tests and plot the force curve vs. the number of cycles.

**Table 3:** Characteristics of the dynamic bending test, specimen 9.54ø × 114 mm

Test feature or detail.	Technical data.	Observations.
Distance between centers L.	90 mm.	-
Test speed (velocity).	5 mm/min.	F1264-03.
Used machine.	Instron 1011, capacity 5 kN.	January 25, 2019 calibration (Mess. Serv. Metrological).
Load amplitude applied.	555 N.	25% Máximum permissible load.
Frequency.	5 Hrz.	Load ratio ≥ 0.1
Cross section of the specimen.	71.48 mm ^2^.	-
Specimen length.	114 mm.	-
Specimen material.	WE43 Casting bars.	Y 3.7-4.3, Zr 0.4-1.0, Gd 0-1.9, Nd 2.0-2.5, Cu ≤ 0.02, Ni ≤ 0.005, Fe ≤0.01, Mg balance
Average weight specimen.	0.01511 kg.	-
Test temperature.	27 °C.	-

The system shown in Fig. 4 was used to perform the 3-point dynamic flexion tests on specimens with a diameter of 9.54 mm and 114 mm in length. This test consists of two supports and a lower base to place the specimen, as well as an upper support mounted on the load cell of the Instron 1011 machine which applies the load during the test to the center of the specimen. A 3D printed polypropylene chamber was prepared to allow the specimen to be immersed in Hank’s balanced salts, modified without phenol red and sodium bicarbonate (H1387-1 L; Batch #SLBG0073), throughout the test. It used a camera system to monitor force vs. the number of test cycles.

The acclimatization treatment that was carried out for a WE43 specimen consists of autoclave sterilization and subsequent immersion in Hank’s solution handled in a chemically inert manner within a closed container using aseptic techniques according to ISO-10993-12. Acclimatization continued for 24 ± 2 h at 37 ± 1 °C. Throughout the test the pH of the Hank’s solution used was not manipulated to avoid any influence on the result, but at the time of its preparation the pH was 7.4. Storage conditions were validated before use.

The incubation of the WE43 specimen in the Hank’s solution was for 24 h, completed under aseptic conditions and in a sterile environment provided by a laminar flow cabinet. Although cells were not incubated in the medium, contamination in the test environment is not impossible due to the interaction of the medium with parts of the Instron machine and fixtures. The possible adhesion of particles and their progression on the surface of the material are effects that should be considered on the result of this test [10].

This acclimatization procedure provides insight for the ISO / TC 194 committee, specifically towards the new changes that are being considered on subsections a (Materials designed to degrade in the body), c (test solution), and e (immersion procedure). This is in light of the modification of the mechanical behavior of this alloy with the interaction of a medium such as Hank’s solution after sterilization [30, 36].

**Fig. 5:**
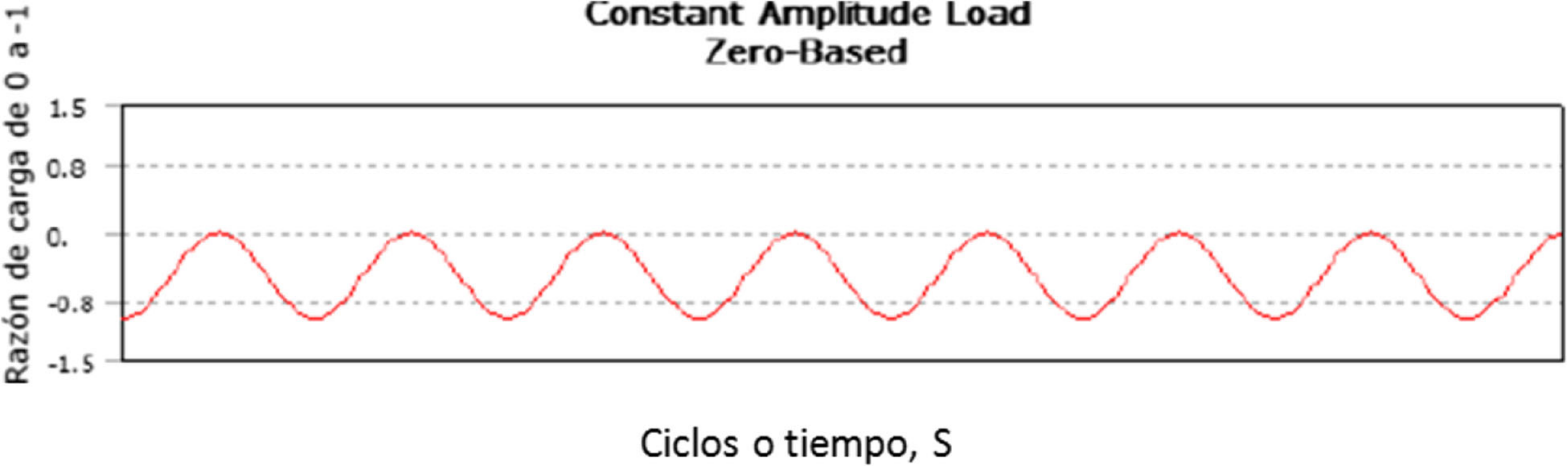
Load ratio from 0 to − 1 vs cycles in time(s)

The characteristics of the system show behavioral data from a dynamic perspective were set at an operating frequency of 5 Hz (5 cycles per second) and the applied load ratio was constant hseno completely compressed. The load applied during the fatigue test was 25% of the maximum permissible load as measured in the simple flex test for each system. This was applied in a period of 0–500,000 cycles. Figure 5 shows a load ratio condition over time, with the application of hseno load and base cycle from 0 to − 1. Qizhi Chen et al., have reported the mechanical working conditions of the human body by defining the frequency per human step at 1 Hz. By biomechanical convention, the operating frequency according to ASTM corresponds to 5 Hz. In their work Qizhi he assumes that a person walks about 1 × 10^7^ cycles in 20 years [32].

### Morphological analysis

Specimens were prepared for microstructural analysis by polishing with 0.3 μm diamond paste to a mirror finish and observation under a scanning electron microscope (SEM; Nanosem200-FEI, Netherlands) equipped with an energy dispersive spectrometer (EDS). To the samples were adhered with a graphite tape to a sample holder to obtain a in an atmosphere under vacuum at a resolution of 50 μm. With this microscope configuration, the initial flashing of the SEM filament was reviewed, selecting HV and emission current window, flash intensity in 2 was confirmed, and the specimen is inserted into the holder, proceeding to the observation. A lamella was prepared for TEM, through a beam of focused gallium ions, thinning the specimen to approximately 60 nm. Subsequently, the sample was kept in a vacuum until the day of the examination and then placed on a sample holder for analysis. A sample for transmission electron microscopy (TEM; Titan 80–300-FEI, Netherlands) was thinned to 60 nm thick by a Double Beam System (FEI QUANTA 200 3D, Netherlands) utilizing Gallium ions before being mounted on a rack and preserved in a vacuum until immediately before observation. The distribution of the elements in the micro-regions and the selected area of electron diffraction (SAED) were performed to help identify the phases. The TEM was operated at 300 keV, opening the condenser lens #2 to 150 with an exposure time of 0.5 s. The SAED in Fig. 11(b) shows a crystalline structure on the diffraction pattern corresponds to the projection of the reciprocal lattice by bright field and dark field. An X-ray diffractometer (XRD; EMPYREAN, Spain), was used for phase identification using a Cu Kα radiation source, with an 8.67 s step-time scan, operated at 45 kV and 40 mA.

### Citotoxicity test

Extracts were obtained by the elution method according to ISO 10993-12:2012. Samples of WE43 were immersed into the cell culture medium for 24 h at 5% CO2, 95% humidity, and 37 °C with a fixed ratio of surface area to medium volume of 1.25 cm^2^/ml [37]. Next, extracts were collected and evaluated for impact in cell viability. For cell culture, a murine fibroblast cell line (L-929; CCL1), was purchased from the American Type Culture Collection (ATCC). Tissue culture medium, fetal bovine serum (FBS), and supplements were purchased from Gibco (IL, USA). Dyes, phosphate-buffered saline (PBS), and dimethyl sulfoxide were procured from Sigma-Aldrich (MO, USA). L-929 fibroblasts were cultured in DMEM with 10% FBS and 2 mM L-glutamine in 5% CO2, 95% humidity and 37 °C. For the Neutral Red Uptake assay (NRU) method, cells were seeded in 96-well plates at a density 1 × 10^5^ cells/well and pre-cultured for 24 h. To allow adaptation of cells before the addition of the extracts.

For the NRU, five non serial dilutions of extracts with culture media over a range from 10 to 100% were placed on cells by quadruplicates. Treatments with standard culture media were sustained as reference (100% viability). ZDC at 0.1% was used as cytotoxic control. After a 48 h incubation period, the 96-well plate was centrifuged (210 g for 10 min) and the medium was replaced by the neutral red stain (100 μl of a 0.2 mg/ml stock solution in cell culture medium) on each well and the plate was re-incubated at 37 °C for 4 h. Afterwards, the plate was washed with PBS once and then removed, allowed to dry for 1 h., and 100 μl of neutral red eluent (Ethanol:dH2O: acetic acid 50:49:1) were added to each well. The plate was then shaken for 1 h to dissolve the dye. After the neutral red had dissolved, the absorbance was measured using a microplate reader (xMark, BioRad, USA) at a wavelength of 540 nm with a reference wavelength of 630 nm. According to ISO 10993-5:2009, a relative metabolic activity of less than 70% was regarded as cytotoxic.

A FEM simulation was performed to visualize the behavior by mechanical integrity of the alloy and its response to failure modes. Ansys 15 software was used, through the workbench module.

## Results

### Mechanical test

**Fig. 6:**
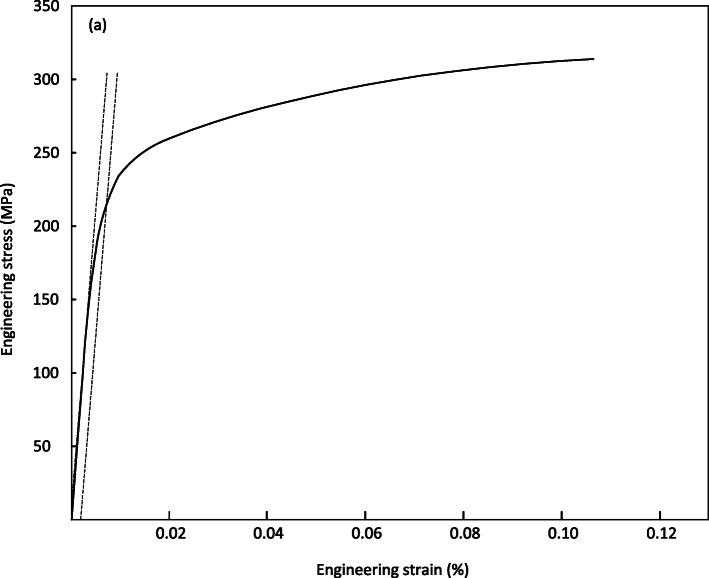
Average stress-strain curve obtained by simple tension. (Offset method)

**Fig. 7:**
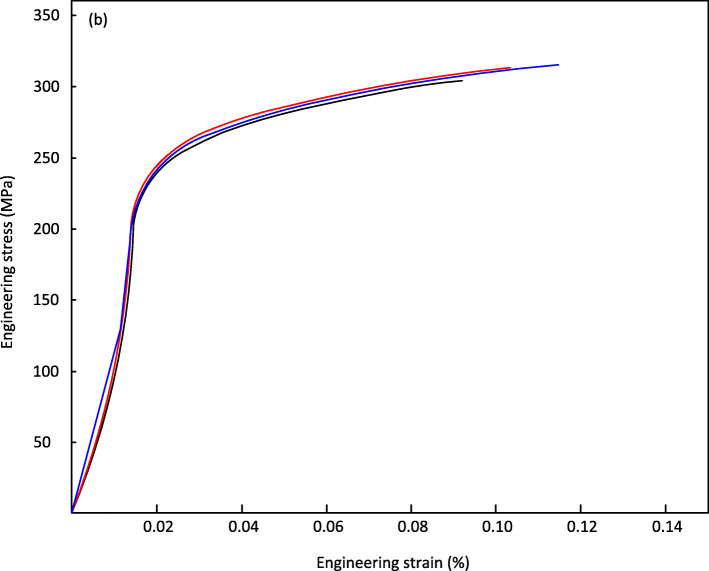
Stress-strain curve of the 3 specimens tested at simple tension

Figures 6 and 7, shows the stress vs. strain curve for the tested material, allowing an analysis of the behavior of the materials to be subjected to the simple stress test referring to 0.2% deformation. Figure 6 shows stability in the three tests with minimal variations in obtaining the average values. Yield strength of 212 MPa was obtained and the tensile strength of the WE43 casting bar material was shown to be 314 MPa with a deformation percentage of 10% and an elastic modulus of 40 GPa. A total of three tests were performed with the specimen supported on the circular conical section rollers as shown in Fig. 3b. The load application was utilizing a cylindrical section roller mounted on the upper support. The load cell was calibrated and tuned, and the load and displacement acquisition system were calibrated. The results of all three tests are shown in Fig. 9, where the maximum forces and displacements are shown.

**Fig. 8:**
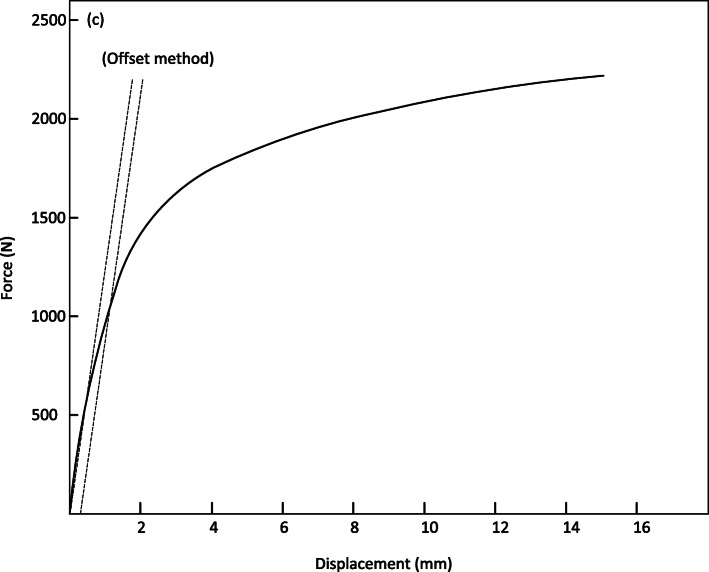
Average values of force vs displacement, simple flexion

**Fig. 9:**
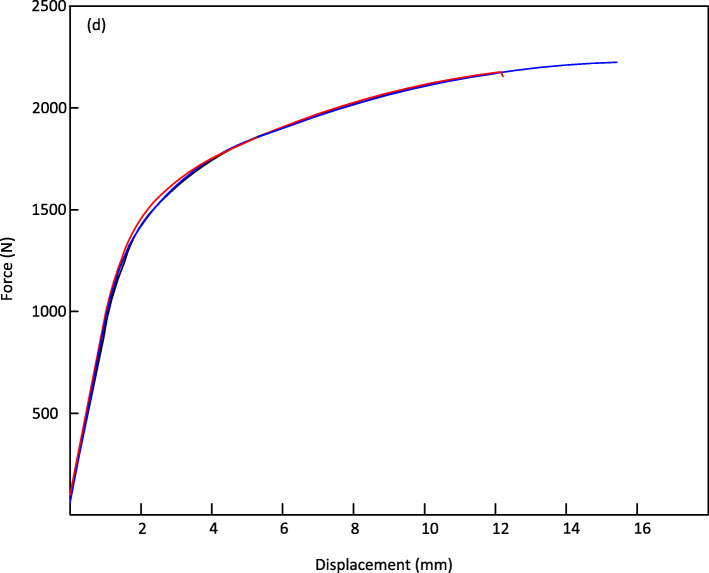
Values of force vs displacement, 3 tests with simple flexion

Figure 8 shows the average values of forces and displacements of the three tests performed on the specimens. In Fig. 8, it can be noted that the maximum force is 2219.77 N, and the corresponding displacement is 15.08 mm. The bending force supported by WE43 specimens of 9.54 X 114 mm results (shown in Figs. 8 and 9) allow us to perform an analysis of the specimen behavior when subjected to 3-point bending.

Figure 9 shows stability in the tests with minimal non-significant variations. For all three tests, there were no fractures or problems in the specimens. According to the ASTM F-1264-03 standard, the force and displacement data were shown to be 1057.14 N and 1.15 mm, respectively. The stiffness of the specimens is 919.13 N/mm. As established by the ASTM F1264–03 standard, the force and momentum data at 0.2% can be calculated to be 1057.14 N and 23.782 N/mm as shown below. Results showed stability with minimal, non-significant variations and no trace of surface fractures.

$$ K=\frac{F}{y}=\frac{1057}{1.15}=919.13\ N/ mm $$

$$ My=\frac{F0.2\%S}{2}=\frac{1057\ast 45}{2}=\mathrm{23,782}\ N/ mm Mmax=\frac{Fmax\ S}{2}=\frac{2219.77\ast 45}{2}=\mathrm{49,994}\ N/ mm $$

$$ El\ e=\frac{s2\left(3L-4s\right)K}{12}=2525\frac{(90)919.13}{12}=13.95\ N/ mm $$

**Fig. 10:**
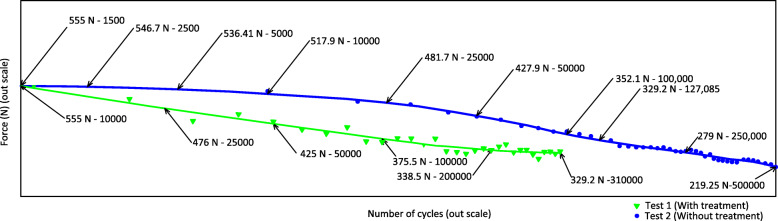
shows the detrimental effect of autoclave sterilization when comparing test 1 (Green) that receives sterilization and acclimatization, against the result of test 2 (Blue) without sterilization, in both tests the protocol of aseptic control and acclimatization.

The results of the two dynamic fatigue flex tests of the system with the device and the specimen subjected to cyclic loading from 0 to − 1 that is from 0 to − 555 N and from 0 to 500,000 cycles are shown in Fig. 10.

By analyzing the results of the dynamic tests with the established conditions, the dynamic behavior of the specimen and the specimen’s degradation time under load are found. If it presented a possible failure or fracture in the specimens answering the above it is possible to establish the quality of the tested element and allow the following analysis.

The results of specimen 1, which was previously subject to an acclimatization and sterilization procedure, presented load amplitude vs. number of cycles where can see that the force has a variation between 555 and 329.2 N during the entire test, corresponding to 310,000 cycles (17 h and 13 min). Thus, the rate of a gradual loss of load in the duration of the test was 225.8 N. By linearizing this graph, throughout the test there is a force reduction rate of 13.11 N / h.

The results of specimen 2 (blue), which was not subjected to sterilization procedure as was specimen 1, but was immersed in Hank’s solution for the test, presented a load amplitude vs. number of cycles, where it can be seen that the force has a variation between 555 and 219.25 N during throughout the test, corresponding to 500,000 cycles (27 h and 46 min), Thus, the rate of gradual loss of load in the duration of the test was 335.75 N. Linearizing the result, specimen 2 had a force reduction rate of 12.09 N / h.

Figure 10 provides a visual comparison of the two tests, highlighting the effect of sterilization on the specimen. The initiation of fatigue cracks occurs internally between the twin’s boundaries and the intermetallic phases, along with the slip bands. However, their propagation is independent of their microstructure [38, 39]. Griffith’s theory indicates that detrimental effects on the mechanical integrity of an Mg alloy are due to the phases [40, 41]. Andrej Atrens et al., have reported that stress cracking can occur below 50% of the yield stress in non-ferrous Mg alloys [36].

The WE43 alloy does not resist prolonged periods concerning a very high number of fatigue cycles this alloy exhibits a prediction behavior of fatigue-life, however this results classifies WE43 with high cycle fatigue regime (HCF) 10^4^ to > 10^6^ [42].

### Morphological analysis

The analysis of the chemical composition, microstructure, and crystallinity of the WE43 alloy concerning the result of mechanical simple tension tests indicates that lattice-Mg HCP have low elongation in the range of 9 to 11%. How an Mg implant is machined depends on the direction of extrusion or rolling of the material to increase the strength of the implant.

**Fig. 11:**
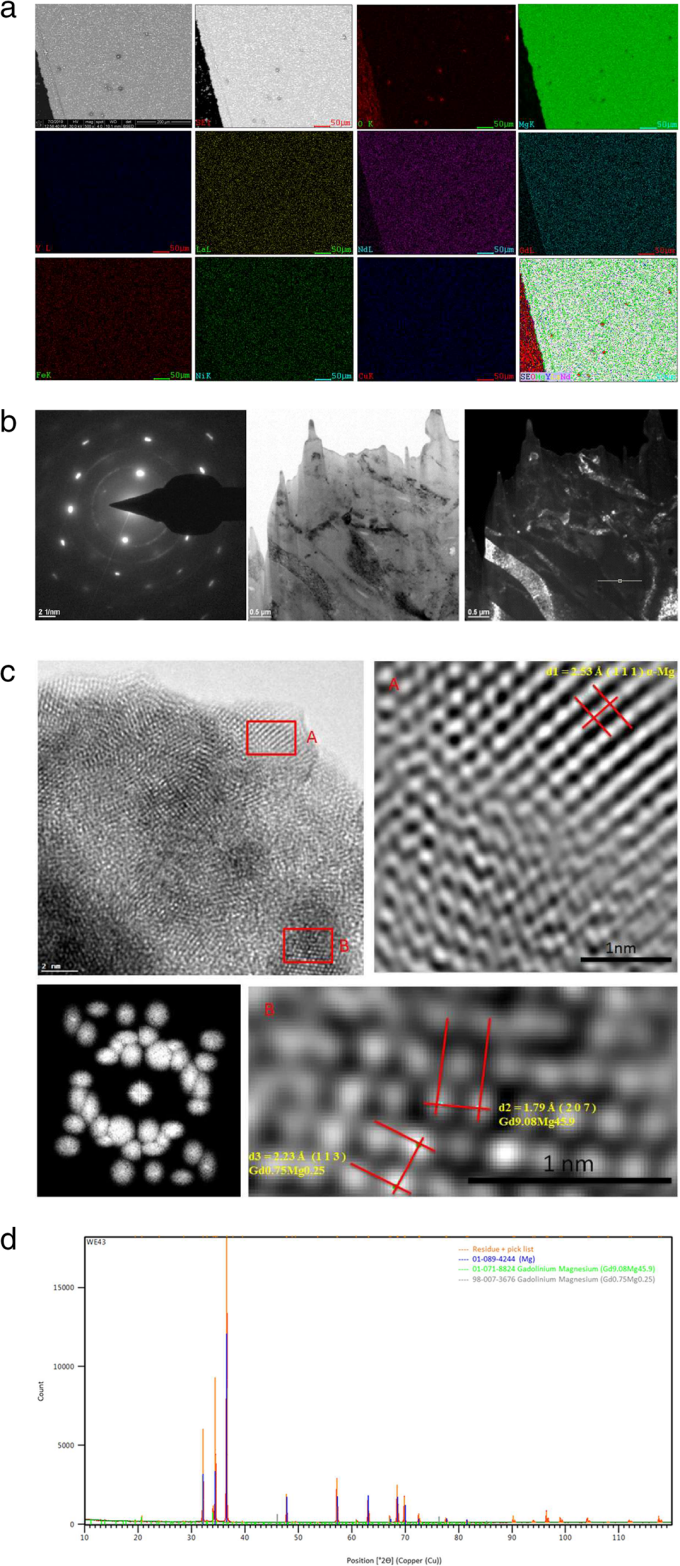
**a** Element mapping by SEM-EDS (O, Mg, Y, La, Nd, Gd, Fe, Ni and Cu) distribution analysis corresponding to those second phase particles. **b** Diffraction pattern image; under examination by TEM (Bright field, dark field). **c** Second phases identified in WE43, α-Mg, Gd9.08 Mg45.9, and Gd0.75 Mg0.25, HRTEM images were taken from specific sites marked with A & B. Lower left image, a cluster of nanoparticles of uniform size and quasi-spherical geometry approximately 5 nm. **d** XRD patterns of the WE43

In general, there is homogenization and positive segregation of the elements in the analysis by SEM-EDS, as shown in Fig. 11(a). The WE43 microstructure obtained by TEM is presented in Fig. 11(b). Images obtained through HRTEM reveal the 0.253 nm, 0.179 nm, and 0.223 nm interplanar spacing, respectively. Figure 11(c) The second phase rich in α-Mg and Gd, Gd9.08 Mg45.9, and Gd0.75 Mg0.25 were detected, Fig. 11(d).

The SAED diffraction patterns and the α-Mg diffraction rings, Gd9.08 Mg45.9, and Gd0.75 Mg0.25 correspond to the second phase that could be appreciated with the help of an XRD analysis. Figure 11(d).

### Citotoxicity results

**Fig. 12:**
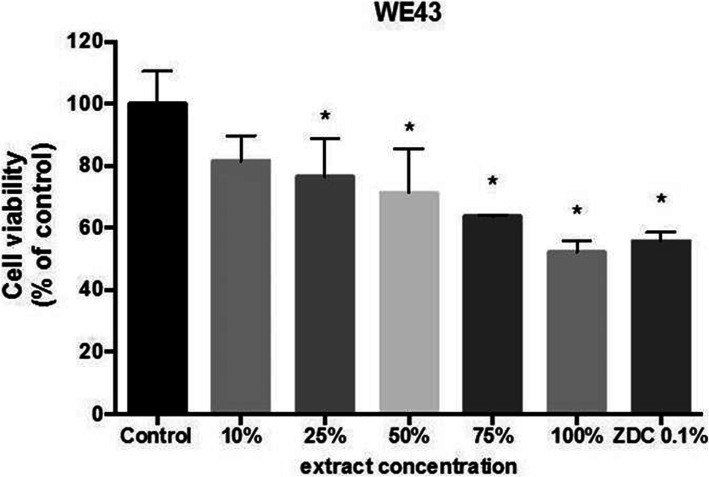
The effect of WE43 extracts in the viability of L-929 murine fibroblasts. The graph represents results obtained after 48 h incubation with non-serial dilutions of WE43 extracts as assayed by the neutral red uptake assay (*n* = 4). Data are expressed as media ± standard deviation in percentage relative to the control cells (treated with cell culture medium). *P ≤ 0.05

As shown in Fig. 12, L-929 murine fibroblasts undergo cell death after 48 h of exposure to incremental concentrations of eluted WE43 extracts in a dose-response manner. The impact of undiluted WE43 extracts on cell viability was comparable to that of cytotoxic molecule zinc diethyldithiocarbamate (ZDC). Cell monolayers were still present, but more than 50% of the cells were lysed or rounded with very evident intracytoplasmic granules and morphological changes and considerable growth inhibition was observed in original undiluted WE43 extracts.

### Finite element method

**Fig. 13:**
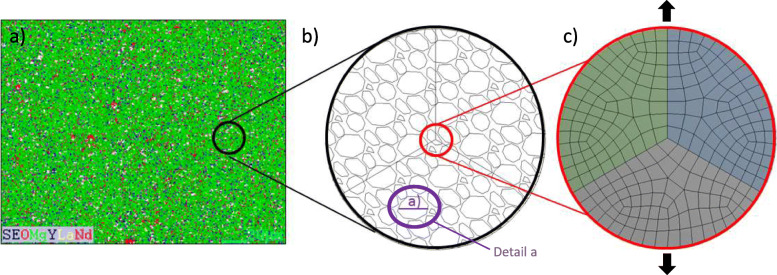
**a** Microstructure. **b** Approach to grain size for an approach to grain boundaries. (Detail **a**) **c** the mesh represents a real microstructure obtained from the examination through SEM-EDS

A simulation was carried out by FEM as a tool to visualize the mechanical integrity of the grain boundaries. For the areas of interest, right at the grain boundaries, a mesh with scale approximate to the grain size of the WE43 alloy is used. See Fig. 13.

One way to achieve optimization could be to relate and identify the direction of the grain; in this work it was characterized by an elongation of the grain horizontally. Figure 13b, detail a) [25].

This alloy is composed of microstructural phases α-Mg, Gd9.08 Mg45.9, and Gd0.75 Mg0.25, with equiaxial grains and twins. WE43 presents homogeneous equiaxial grains, with a recrystallized microstructure and twins inside the equiaxial grains. In Fig. 13b, the direction of elongation of the equiaxial grains obtained from a WE43 bar is also presented. Mg-based alloys are mainly composed of an alpha primary phase and second phases distributed along the grain boundaries that depend on the amount of alloying elements added in the alloy formulation [43].

**Fig. 14:**
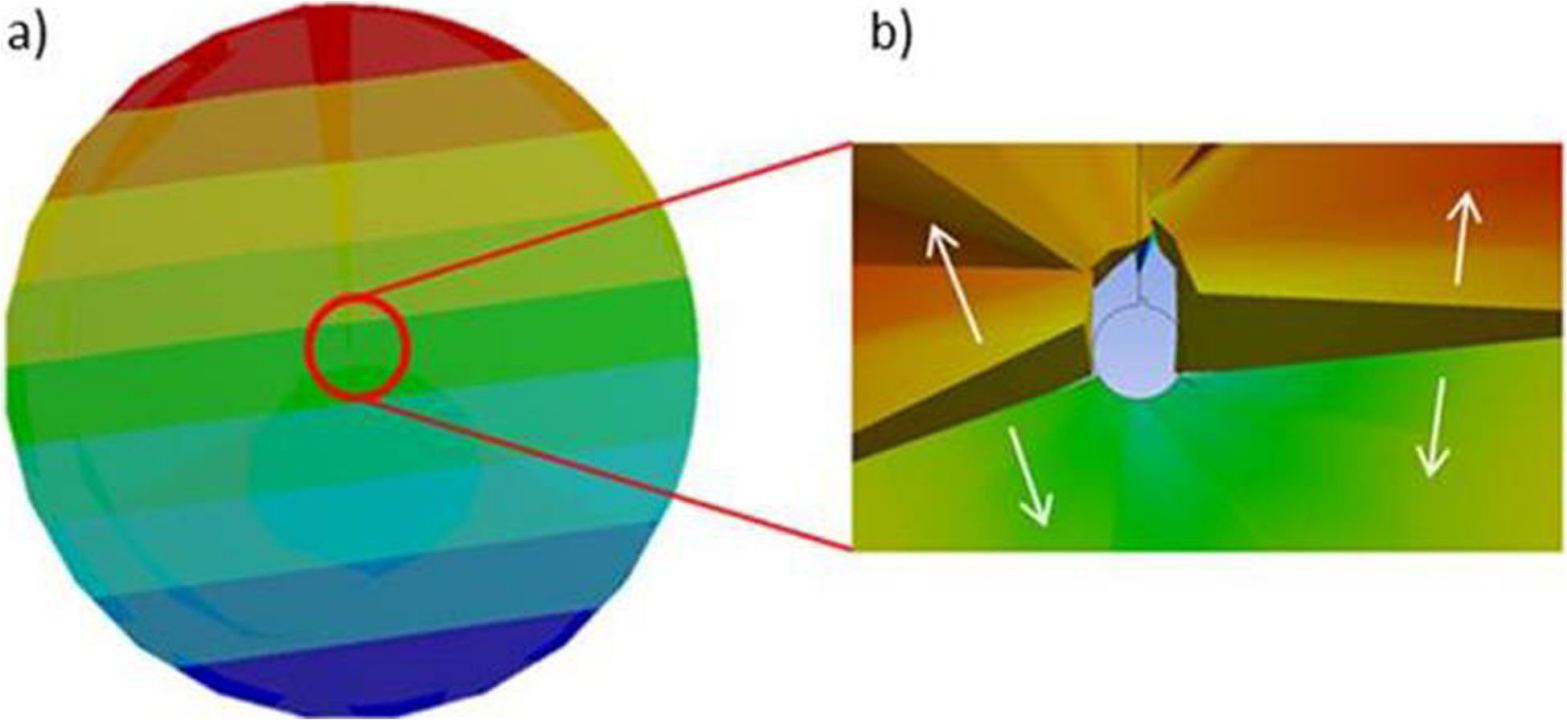
**a** Directional deformation. **b** Displacement of elements (grains)

The directional deformation along the grain boundaries is highlighted, caused by the force acting in tension, causing dislocation of the grains. See Fig. 14.

**Fig. 15:**
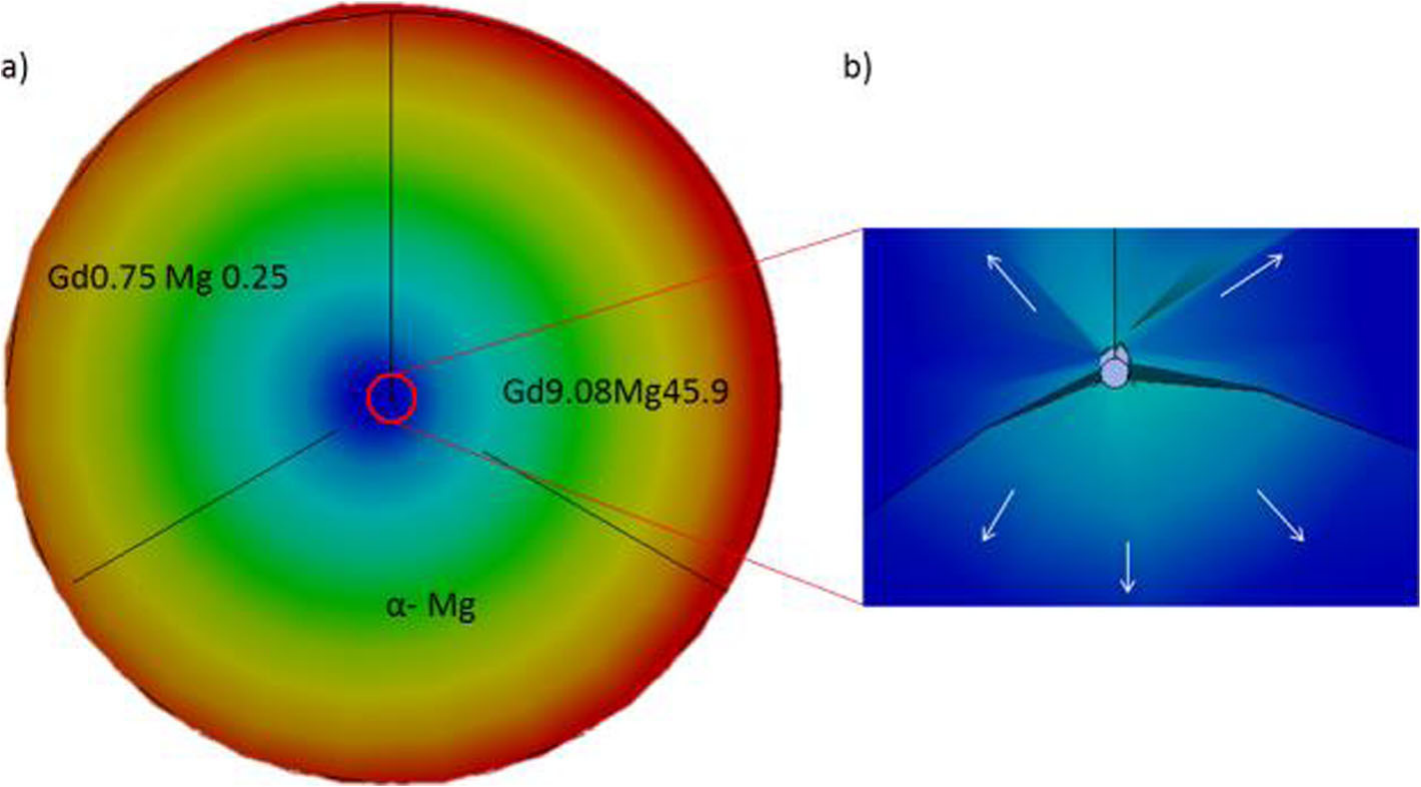
**a** Deformation concentration towards grain boundaries. **b** Equivalent elastic deformation stimulating the propagation of the cracks

The total deformation of a WE43 alloy will be concentrated towards the areas where the grain boundaries of the different phases converge, causing cracks to start and also reducing ductility [44–47]. The equivalent elastic deformation stimulates the propagation of these cracks from the grain borders to the outside. In this simulation, the 3-grain boundaries corresponding to each phase are hypothesized (Fig. 15a). The rate of propagation of a crack implies the growth of the crack due to the applied effort, starting at small values like 10^− 8^ mm/cycle, gradually increasing and concentrating the effort at the site where the nucleation begins (Fig. 15b).

The equivalent effort (Von-Mises) causes moments at the grain boundaries, starting the propagation of a crack by debridement of elements (grains). The total equivalent deformation acts from the inside of the grain boundaries to the outside. The effort is mainly concentrated on the segregations of material, weakening the mechanical behavior of the WE43 alloy. The relative orientation of the grains and crystals concerning the form of loading, in addition to their direction, create stress concentrators and crack initiators at the grain boundaries. Anisotropy and elastic properties of a WE43 alloy are related to the shear modulus of the material, which will affect the plastic deformation and the movement of dislocations. The mechanism of plastic deformation between the grains will present a high density in the dislocations, and this could lead to the initiation of inter-crystalline cracks.

The flexural behavior of the WE43 alloy is not a critical factor according to experimentation because there are no observable fractures. These results make it reasonable to establish that, for the WE43 alloy obtained by casting bar, determining the orientation of the grain, and relating it to manufacturing procedures could be an effective method to optimize its mechanical properties. The present work considered the following identification to the orientation of the grain, elongation of the grain horizontally. Figure 13(b), detail a.

There is no macroscopic indication warning of potential failure in a fatigue test, unlike the stress test, a material may fail due to an applied stress amplitude even being below the yield strength. This could be attributed to the debridement of the atoms in its equilibrium zone to the residual stresses of the material and even to the concentration of stresses or impurities present at the time of melting.

The behavior of the WE43 material is established and adapted to the recovery period of a tibial shaft fracture as a starting point to idealize the optimal recovery point of a fracture vs. implant integrity, curve of the optimal point of the material adapted to degradation time and stress [48].

## Discussion

An implant made of WE43 alloy could be used in trauma applications as an intramedullary nail [49], with the following considerations. The mechanical properties of the alloy WE43 as-cast bar are very close to the mechanical properties of the cortical bone that is in the anatomical area of the tibia shaft. It is extremely beneficial that the elastic modules are close (a difference of just 17 GPa), as this will help to avoid bone deformation problems from variations in the loads that the bone will support. The theory of neoformation proposed by Frost suggests that bone remodeling could prevent the accumulation of fatigue damage in the bone through a controlled sequence of activation, resorption, and formation through a multicellular bone unit. In addition, the sterilized implant suffers a decrease in the number of cycles, and therefore a decreased lifetime in vivo. This suggests a detrimental effect of sterilization. Comparing the fatigue test results of a sterile and an unsterilized specimen provides evidence that the behavior of this alloy is significantly altered by sterilization. In a curve that describes the amount of force that an implant would be resisting with the diameter and length corresponding to an IIM, what which could be used to relate in clinical practice to the periods in which stimuli are allowed to apply for the rehabilitation stages [50, 51].

The difference in the elongation of 7% of WE43 concerning cortical bone is beneficial because it will allow the bone to flex naturally with just a slight resistance that will control the micro-macro movements of the fracture hematoma area, which will benefit the period of recovery of the fracture. An implant manufactured with WE43 will withstand greater stress than cortical bone by 12% which will minimize shear loads. Furthermore, due to the elastic limit the WE43 implant will support 84% in load more than bone.

In the fatigue test, for specimen 1, an acclimatization and sterilization treatments were used while for specimen 2 only the acclimatization treatment was used. Compared to specimen 1, specimen 2 could sustain 190,000 additional cycles, indicating that the sterilization method is related to causing rapid degradation.

For specimen 2, which was not subjected to a sterilization procedure, reached 329.2 N at 127,085 cycles (7 h and 3 min), corresponding to a 40% increase in the duration of the test compared to specimen 1. This establishes a clear relation between sterilization and the extension of the life of the specimen. This provides quantitative evidence regarding the importance of preventing Mg from being exposed to a body medium, and an effort must be made to increase the life of the coatings that are applied to future alloys. The reduction of the strength in an alloy of Mg WE43 immersed in Hank’s solution is relevant because it characterizes the mechanical behavior and the degradation of the material. Combining these parameters with the recovery time of a fracture in the tibial shaft and the time it takes for material degradation to occur creates a reference point on how much force the implant would stop supporting depending on the stage of healing of the fracture. The accumulation of structural damage on the WE43 implant material is not characterized by a uniform cyclic stress condition, so the Wöhler S/N diagrams do not adapt to how the implant will be loaded during the time in service. Distribution of the load against the number of cycles will help us identify the load ranges in the time during which the degradation of the material occurs.

WE43 has a recrystallized microstructure with second homogeneous phases in the matrix, where equiaxial grains with internal twins are presented. Notably, the resistance of this alloy is very similar to cortical bone (approximately 34 MPa apart). In addition to the analysis by SEM-EDS secondary phases were identified by XRD. It is suggested to improve the resistance of this alloy by refining the microstructure. The twins inside of the grains could prevent the dislocation of bands, as the twins are related to the yield of the material and the phases of Gd normalize the Mg matrix, which can prevent accelerated degradation [44, 52, 53]. Homogeneity in the microstructure of the alloy is shown with traces of pitting corrosion, indicating it is resistant to corrosion. However, when the pitting occurs it acts mechanically as a stress concentrator facilitating rapid corrosion and affecting mechanical properties and speeding up the degradation of the alloy [38, 54, 55]. For this application, the use of a coating which slows down the appearance of pitting, it is a multidisciplinary strategy that will depend entirely on the initial weeks in which it is required to maintain the mechanical integrity of the fully integrated implant. It is suggested to evaluate a coating that allows keeping the surface of the implant intact for 5 weeks, to minimize the places where the propagation of a crack may begin.

It is advisable to add elements such as Y, Zr, and Nd at low levels to refine the microstructure and reduce degradation [56, 57]. The nucleation begins at the origin of pitting corrosion, in the formation of cracks due to fatigue loading, and its subsequent propagation occurs through the sliding bands internally through the twins and along the grain boundaries. The distribution of mechanical stresses will not be homogeneous through the material due to crystallographic orientation of second phases and the generation of precipitates and pores, some of which are produced by pitting corrosion. Positive segregation of the elements of the casting process was observed, the orientation of the grain in an Mg implant has a remarkable relationship in the mechanical properties [58]. The crystalline HCP structure of the WE43 alloy has a mechanical behavior that is represented in the curves and values obtained from the characterization shown. Positive segregation is a highly desired attribute for elements like yttrium and Nickel.

The crystalline structure Mg HCP is related to isotropic multilinear behavior. The preferential orientation of the grain suggested horizontally, is an ideal condition that according to the simulation by finite element would optimize the mechanical properties of the alloy when subjected to tension and flexion. However, the preferential grain orientation of a WE43 alloy is characterized according to the orientation of its planes. The orientation of the grain is vital to optimizing the mechanical integrity of the implant. The horizontal orientation, as was utilized throughout this experiment, shows evidence of how the mechanical properties are superior to those normalized by convention.

The negative impact on adhered L-929 cells exposed to WE43 (evidenced by low levels shown of cell metabolic activity/ viability) has limited the approval of WE43 alloys for MD. However, WE43 has been permitted for use in clinical applications by regulatory agencies in Europe. Using WE43 as a base, as long as the yttrium and nickel are controlled microstructurally and kept in low percentages to avoid toxicological problems, this has allowed the medical industry advances in the development of clinical test protocols, which allow the evaluation of long-term biosafety [59–61]. The effect of yttrium and nickel on cellular interaction has been shown to reduce the biological potential of WE43, as the toxicity of these elements affects cell viability and its biological environment. However, if they are kept at the lower limits of their chemical composition and the degradation rate in vivo is slowed down by a coating which helps to dose its dissolution, the result of the cellular viability would be positively different, as implied in the approvals that are expected in the revision of ISO 10993-15.

A strategy to use WE43 alloys in the manufacture of MD, and specifically IIM, would be to keep elements such as yttrium within the minimum allowed range. Additionally, it would be beneficial to control the casting process to avoid contamination of elements such as nickel, which must be less than or equal to 0.005% by weight. The process of segregating elements that occurs after smelting should resolve the issue of homogeneous dispersion and preferably avoid the agglomerations of these elements. Manufacturing an IIM based on the WE43 alloy should consider allowable intake dose that the human body can tolerate for yttrium and nickel. Gradual release to the body due to degradation of the Mg matrix in small doses triggers pathophysiological and toxicological responses. Although, not exceeding the limits as suggested in does not guarantee to avoid overdose, the values of dissolved elements in blood serum level have experimental exposure limits of yttrium: < 47 μg and nickel: 0.05–0.23 lg L-^1^. Acceptance criteria for the use of the WE43 Mg base alloy and any adverse effects that may arise should be considered.

## Conclusion

It can be said that the stress and strain tests for the three specimens of the WE43 casting bar material, under the loading conditions applied according to the ASTM E8–04 standard, yielded satisfactory results considering the yield strength, ultimate tensile strength, percent deformation, and elastic modulus of the WE43 specimens. Furthermore, the tests determining strength for simple flexion in three points on three specimens under the load conditions applied according to ASTM F1264–03 A1 were satisfactory as appropriate maximum loads, displacements, structural behavior, stiffness, and elastic limits were observed.

1. Because decreasing the exposure of Mg in vivo can increase the time that an Mg-based material would function within the organism, carefully selecting the method of sterilization on coatings will be a key strategy in the reducing degradation. As degradation is reduced, Mg-based materials are more likely to become accepted by regulatory agencies.
2. The performance of a fatigued WE43 alloy could be improved for use in implants of the intramedullary by improving the microstructure. Homogenizing the alloy and making fine and small grains through the grinding of elements and minerals allows easy dispersion contributing to positive segregation during the smelting process, which be must be regularly monitored.
3. The choice of coating for any material is very important depending on medical application; the coating used for Mg alloys will determine the time that the alloy will provide mechanical resistance to a fracture.
4. A WE43 implant can withstand a test of 5 × 10^5^ cycles. This is the same standard of traditional biomaterials as classified in the HCF regime. However, it should be noted that titanium or stainless-steel based implants can, in comparison, withstand 1 × 10^6^ cycles. These classic biomaterials which support this high number of cycles can remain in vivo for up to 30 years in the body of patients because the removal of them causes more complications. Indeed, the decrease in longevity of an implant manufactured in WE43 in 50% in fatigue-life prediction raises more than one question for the scientific community. However, it should be considered that the WE43 implant should only remain mechanically intact during the period of consolidation of the fracture (approximately 6 months). After approximately 16 months, the WE43 will be almost completely excreted by the biological mechanisms of the human body.
5. This work establishes that the orientation of the grain must be horizontal to the load, which allows optimal increasing of the resistance, as shown by the FEM simulation representing the fracture behavior of the alloy.
6. L-929 murine fibroblasts experienced a cytotoxic outcome in a dose-response fashion when exposed for 24 h or 48 h to incremental concentrations of original extracts of WE43. The effect of WE43 extract on cell viability was comparable to that of cytotoxic molecule zinc diethyldithiocarbamate. Cell cultures exposed to WE43 extracts are still capable of cell adhesion, but there are very evident intracytoplasmic granules and morphological changes, with occasional cell lysis. More than 50% of growth inhibition is observed.
7. It is suggested to evaluate the gamma radiation sterilization method, which could reduce the detrimental effect on Mg alloys.

## Data Availability

OK

## Abbreviations

IIM: Intramedullary Implant
Mg: Magnesium
REEs: Rare earth elements
MDs: Medical devices
KFDA: Korean Food and Drug Administration
ASTM: American Society of Testing Materials
FEM: Finite element method
